# Scaffolds Based on Silk Fibroin with Decellularized Rat Liver Microparticles: Investigation of the Structure, Biological Properties and Regenerative Potential for Skin Wound Healing

**DOI:** 10.3390/pharmaceutics14112313

**Published:** 2022-10-27

**Authors:** Maria Bobrova, Liubov Safonova, Anton Efimov, Alexey Lyundup, Natalya Mozheiko, Olga Agapova, Igor Agapov

**Affiliations:** 1 Academician V.I. Shumakov National Medical Research Center of Transplantology and Artificial Organs, Ministry of Health of the Russian Federation, 1 Shchukinskaya Street, 123182 Moscow, Russia; 2 Educational Resource Center for Cellular Technologies, Peoples’ Friendship University of Russia (RUDN University), 6 Miklukho-Maklaya Street, 117198 Moscow, Russia

**Keywords:** liver decellularization, extracellular matrix, silk fibroin, biocompatible polymers, regenerative potential, scanning probe nanotomography, full-thickness skin wound

## Abstract

The development of advanced biomaterials and constructs for accelerated recovery of damaged tissues is a key direction in regenerative medicine. Biocompatible scaffolds based on natural biopolymers are widely used for these tasks. Organ decellularization enables obtaining a cell-free extracellular matrix (ECM) with preserved composition and biological activity. The objectives of the present work were combining these two approaches for the development of a composite scaffold based on silk fibroin and ECM microparticles and assessing its structure, biological properties, and regenerative potential. ECM microparticles were obtained by grinding the decellularized matrix of Wistar rat liver in liquid nitrogen. Scaffolds in the form of films were prepared by the casting method. The sinuous and rough topography of the scaffold surface was assessed by the scanning probe nanotomography (SPNT) technique. The inclusion of ECM microparticles in the composition did not affect the elasticity and tensile strength of the scaffolds. The obtained scaffold was non-toxic to cells, maintained high levels of adhesion and proliferation of mouse 3T3 fibroblast and Hep-G2 cells, and showed high regenerative potential, which was studied in the experimental model of full-thickness rat skin wound healing. The wound healing was accelerated by 1.74 times in comparison with the control.

## 1. Introduction

The primary tasks of tissue engineering and regenerative medicine are manufacturing scaffolds, imitating the microstructure of the native extracellular matrix of tissues and organs, and recreating a favorable environment for cell adhesion and proliferation. At the same time, an important task is to develop and create constructions with not only the necessary biochemical, immunological, and mechanical properties for cell adhesion, cell-to-cell communication, and their production of the extracellular matrix in vivo, but also a defined three-dimensional micro- and nanoscale morphology. Commercial medical implants made of synthetic materials have some advantages (including improved mechanical properties), but the methods for obtaining such implants do not allow repeating the necessary architecture, micro- and nanostructural features, and functional activity of native tissues. This problem can be solved by the use in regenerative medicine of biological grafts that include components of native tissue.

Decellularized tissues and organs may serve as sources of cell-free ECM, which is used for such regenerative medicine tasks as (1) fabrication of specific microcarriers for cells, (2) fabrication of hydrogels from lyophilized decellularized tissue treated with pepsin, followed by dissolving in phosphate-buffered saline, and (3) fabrication of coatings from a solution of the components of the extracellular matrix obtained from lyophilized decellularized tissue [1].

Microcarriers can be used as supporting material in transplantation in dry form or in the form of suspensions for injection as a minimally invasive method of therapy. For example, microparticles from decellularized skin tissue were used to regenerate the skin [2].

The traditionally used hepatocyte transplantation has practical limitations, mainly owing to the difficulties associated with obtaining of a sufficient number of functioning hepatocytes necessary for therapeutic efficacy [3]. In addition, primary hepatocytes easily lose their viability and their function in culture both in vitro and after transplantation [4]. One solution to these problems is the culture of hepatocytes on a liver extracellular matrix hydrogel or on the coating of surfaces of artificial carriers with components of the liver matrix for subsequent culture and transplantation of hepatocytes [5,6]. The components of the extracellular matrix in the composition of the constructions create a natural microenvironment favorable for hepatocytes, similar to the microenvironment of hepatocytes in native tissue. Such functional carriers are being developed for liver tissue engineering, cell therapy, and transplantation. Moreover, coatings of decellularized liver tissue and a hydrogel based on it have been shown to provide more efficient adhesion and proliferation of hepatocytes compared with classical matrices such as commercial collagen and Matrigel [7].

In reconstructive surgery, it is often necessary to restore adipose tissue in the event of postoperative, congenital, or post-traumatic loss, which can lead to scar tissue formation or loss of function. The literature describes the use of decellularized human greater omentum from cadaveric donors [8], as well as omentum of pigs [9]. The greater omentum is used in various surgical procedures owing to its high degree of vascularization, high angiogenic activity, and high production of growth factors. The decellularization of the omentum was conducted according to many protocols, using physical, chemical [10,11], and enzymatic methods [12,13,14], as well as their combinations [15,16,17]. The obtained extracellular matrix is a porous complex three-dimensional structure of collagen and elastic fibers, which includes the vascular network. Decellularized adipose tissue is not toxic to cells, supports high levels of their adhesion and proliferation, reconstructs a microenvironment for adipogenesis, angiogenesis and cell infiltration, and shows no signs of inflammation and rejection during subcutaneous implantation [18].

The obtained decellularized adipose tissue has been used as a material for various products: hydrogel scaffolds based on decellularized tissue [19] and composite scaffolds in combination with chitosan [20], chondroitin sulfate [21], silk fibroin [22], polyethylene glycol [23]; injected microparticles of decellularized tissue [24]; microcarriers from the extracellular matrix [25] and in a composite with alginate [26], lyophilized microparticles of decellularized tissue [27]. In addition, the potential of decellularized adipose tissue as a universal material for creating personalized products for regenerative medicine using a 3D printer was described [28]. In this case, adipose tissue was used as a source of extracellular matrix for the hydrogel and as a source of stem cells, which will subsequently vitalize the printed scaffold. One of the advantages of this approach is the creation of an anatomically and biochemically personalized non-immunogenic graft.

According to the literature, decellularized myocardial tissue has also been used for the manufacture of hydrogels. After grinding the extracellular matrix, the decellularized tissue was treated with pepsin, and the self-organizing hydrogel with preserved bioactivity was obtained. This was demonstrated in vitro using a culture of mouse fibroblasts HL-1, where the extracellular matrix maintained high metabolic activity and cell proliferation. In an ischemia simulation experiment, microparticles of decellularized heart tissue had a specific cell protector effect on cardiomyocytes [29].

Pancreas transplantation is the only reliable long-term therapy for patients with insulin-dependent diabetes mellitus. Since the incidence of diabetes mellitus among adults and children is growing every year, and at the same time there are a number of problems with transplantation, such as a lack of donor organs, the high cost of transplantation and subsequent maintenance immunosuppressive therapy, the necessity to develop new approaches to create tissue-engineered constructions for the treatment of pancreatic diseases is more relevant than ever. Decellularization of the human pancreas was performed by perfusion with a solution of Triton X-100 and ammonium hydroxide or by treatment with deoxycholate. Furthermore, the obtained extracellular matrix was lyophilized and subjected to cleavage in a solution of pepsin in hydrochloric acid, as a result of which a hydrogel of the pancreatic extracellular matrix was obtained. The obtained hydrogel was recellularized with various types of pancreatic cells and showed biocompatibility with the insulinoma cell line, beta-like stem cells, and endothelial cells, which can subsequently contribute to adequate graft vascularization. The absence of immunogenicity of the obtained hydrogel was also demonstrated in vivo in mice [30].

The decellularization method was also applied to the human placenta, owing to the high content of extracellular matrix components and endogenous growth factors [31]. Cells were removed by perfusion of solutions of the detergents sodium dodecyl sulfate and Triton X-100 [32]. It should be noted that an extensive vascular network was preserved in the obtained extracellular matrix, which will ensure adequate delivery of nutrients and oxygen to cells after vitalization [33]. The regenerative potential of the decellularized placenta as a coating for the healing of a full-thickness skin wound was investigated. In this case, decellularization was conducted by homogenizing the tissue, and then a complex of physical, chemical, and enzymatic treatments was applied to remove the cells. After that, thin scaffolds were formed from the extracellular matrix and subjected to freeze-drying. The obtained scaffolds were studied in vivo in a rat skin wound healing model. This material was effective because of its anti-inflammatory and antibacterial properties and low immunogenicity [34].

Many experimental data on the possibility of using decellularized tissue in various fields of regenerative medicine, as well as on the study of the unique biological properties of the extracellular matrix, determine the relevance of the development of alternative methods of decellularization for the creation of grafts for liver regeneration. The goal of the present work is the development of a novel kind of biocomposite material combining silk fibroin, as a base biopolymer, and ECM microparticles. We report on the development of composite scaffolds based on silk fibroin with microparticles of decellularized rat liver tissue, investigation of its structural and biological properties, and in vivo assessment of its regenerative potential in a rat skin wound healing model.

## 2. Materials and Methods

### 2.1. Animals

We used male Wistar rats (250–350 g) both for liver explantation with consequent decellularization and for investigation of the scaffold regenerative potential in a model of full-thickness skin wound healing. All experiments with animals were approved by the Local Ethical Committee of the V.I. Shumakov National Medical Research Center of Transplantology and Artificial Organs. Experiments were conducted in accordance with Directive 210/63/EU of the European Convention for the Protection of Vertebrate Animals Used for Experimental and other Scientific Purposes (1986, ETS 123).

### 2.2. Decellularization of Liver

Hepatic portal vein cannulation was performed with the use of a Vasofix Certo 18 G catheter (B. Braun, Hessen, Germany). After that, the liver was explanted from the rat organism, and a perfusion pump (B. Braun, Germany) was used to remove the blood from the organ; the liver was perfused with 200 mL of phosphate-buffered saline (PBS) (Sigma-Aldrich, St. Louis, MO, USA). Decellularization was conducted by sequential 3-stage perfusion of the explanted liver with solutions of 0.1% sodium dodecyl sulfate (Dia-M, Moscow, Russia) in PBS (500 mL) with increasing concentrations of Triton X-100 (Sigma-Aldrich, St. Louis, MO, USA): 1%, 2% and 3%. The organ was perfused with these detergent solutions over 72 h. Then, to remove the detergents contained in the solutions from the liver, it was perfused with PBS. A perfusion rate of 150 mL/h was used in all stages. The obtained decellularized liver was stored at 4 °C until use [35].

### 2.3. Fabrication of Microparticles from Decellularized Liver

Decellularized rat liver tissue was ground with surgical scissors, transferred to a tube, and the volume was adjusted to 15 mL with a solution of 15% glycerol in PBS (pH = 7.4). Then tissue was incubated for 20 min and centrifuged for 10 min at 8500× *g*. The precipitate was milled in liquid nitrogen using a pre-cooled pestle and mortar for 15 min. The obtained particles were transferred to a clean pre-cooled tube, and the volume was adjusted to 35 mL with distilled water with stirring.

After subsidence of the obtained suspension of particles during the day, the upper 1/3 of the suspension of settled particles was taken, which was centrifuged for 10 min at 1355× *g* twice. Then the supernatant was centrifuged for 10 min at 720× *g*. The obtained supernatant was centrifuged for 10 min at 12,100× *g* 4 times. After that, the supernatant was taken completely, and the obtained precipitate was diluted in double-distilled water in a volume 10 times smaller than the volume of the selected suspension of particles after grinding in liquid nitrogen.

### 2.4. Fabrication of Silk-Fibroin Film Scaffolds

The composite scaffolds in the form of films were obtained using the casting method [36]. Silk fibroin was obtained from silk threads of the *Bombyx mori* silkworm. In the first stage, the threads were cut into 5 cm fragments and purified from sericin. For this, 1 g of threads was boiled in a 500 mL of 2.52 M sodium bicarbonate aqueous solution in a water bath for 40 min and washed with 3.6 L of water. Then, the silk was boiled in 500 mL of water in a water bath for 30 min and washed with 3.6 L of distilled water. The last procedure was repeated 3 times. The purified silk fibroin was dried at room temperature.

An aqueous solution of fibroin (1 mL) was prepared by boiling a solution of 389 mg of calcium chloride, 388 μL 96% ethanol, 544 μL double-distilled water, and 130 mg of silk fibroin for 5 h in a water bath [26,27]. Then, the solution was centrifuged for 7 min at 12,100× *g*. The supernatant was dialyzed against double-distilled water and centrifuged for 7 min at 12,100× *g*. The fibroin solution concentration was measured with a Thermo Genesis 10 UV spectrophotometer (Thermo Fisher Scientific, Waltham, MA, USA) at a 280 nm wavelength (molar extinction coefficient of fibroin 1.07). To obtain a film with a diameter of 1 cm^2^, 100 μL of the working silk fibroin solution with a concentration of 20 mg/mL was deposited on the surface of the polished Teflon and dried for 2 days at room temperature. The films were incubated in 96% ethanol for 15 min and were separated by scalpel [36].

For the fabrication of the composite scaffolds based on silk fibroin in the form of films, a suspension of microparticles of decellularized rat liver tissue was added to a working solution of silk fibroin. Before adding, the suspension was carefully dispersed and added to the solution for fabricating a scaffold to a final concentration of microparticles of 8 mg/mL.

### 2.5. Mechanical Characteristics of Obtained Scaffolds

Mechanical characteristics of fabricated scaffolds were studied using a Zwick/Roell BZ 2.5/TNIS testing machine (Zwick GmbH & Co. KG, Ulm, Germany). There were two groups of samples: group 1—scaffolds in the form of films based on silk fibroin, group 2—composite scaffolds in the form of films based on silk fibroin with microparticles of decellularized rat liver tissue. Samples with dimensions of 1.5 × 5.0 cm were prepared for testing. Mechanical measurements were performed at a speed of 50 mm/min with preload of 0.05 N. As a result, two basic characteristics were determined for each sample: elasticity or elongation as a percentage of the initial length of the sample and tensile strength (MPa). Statistical processing of the data obtained was conducted using the TestXpert software version III (Zwick Roell GmbH & Co. KG, Ulm, Germany).

### 2.6. Scanning Electron Microscopy (SEM)

Samples for SEM were fixed in a solution of 2.5% glutaraldehyde (Sigma-Aldrich, St. Louis, MO, USA) in PBS for 2 h in the dark at 4 °C. Fixed samples were washed 5 times for 5 min in PBS and dehydrated by incubation in ethanol solutions with increasing concentrations (10%–30%–50%–70%–80%–96%) for 30 min in each solution. Then, samples were transferred into acetone. Before the measurements, critical point transition drying of the samples was conducted with a K850 Critical point dryer (Quorum Technologies, Lewes, UK). A 10 nm thick layer of gold was deposited on the dried samples at an ion current of 20 mA in argon atmosphere (pressure: 1 mbar) with use of a Q150R ES (Quorum Technologies, Lewes, UK) vacuum spraying device.

SEM analysis of the prepared samples was conducted using a Tescan Vega 3 (Tescan, Brno, Czech Republic) scanning electron microscope. Images were processed by Tescan VegaTC software version 4.2.17.0 (Tescan, Brno, Czech Republic) [35].

### 2.7. Scanning Probe Nanotomography

The three-dimensional structure of fabricated scaffolds was studied by the scanning probe nanotomography (SPNT) technique. This method utilizes a specialized integrated SPM/ultramicrotome apparatus combining a scanning probe microscope and an ultramicrotome (Leica EM UC6, Leica Microsystems GmbH, Vienna, Austria). It enables acquiring SPM data from the sample blockface surface immediately after ultrathin sectioning by an ultramicrotome diamond knife [37].

Samples for SPNT analysis were fixed, dehydrated, and embedded in epoxy embedding medium (Sigma-Aldrich, USA) using a protocol described in [35]. Consecutive ultramicrotomy sectioning of the samples was conducted using an ultrasonic diamond knife Ultrasonic 35 (Diatome AG, Nidau, Switzerland) with 3.0 mm blade width. After each 150 nm-thick section, consequent semicontact mode SPM measurement of the freshly sectioned blockface surface was performed at a scanning speed of 1 Hz. Silicon cantilever probes ETALON HA_HR (Tipsnano OÜ, Tallinn, Estonia) with a tip radius of curvature <10 nm and a resonant frequency of 390 kHz were used for SPM measurements. The three-dimensional sample structure was reconstructed and visualized by integration of a series of tomographic SPM images with the Image Pro AMS 6.0 software package using the 3DConstructor option (MediaCybernetics Inc., Rockville, MD, USA).

### 2.8. Cytotoxicity Assay

Mouse 3T3 fibroblasts were utilized for cytotoxicity analysis performed using the MTT test. Cells were cultured in 300 μL of Dulbecco’s Modified Eagle’s Medium (DMEM) with low-glucose concentration (PanEco, Moscow, Russia) containing 10% fetal bovine serum (HyClone, Logan, UT USA), 40 μg/mL gentamicin (Sigma-Aldrich, St. Louis, MO, USA), and 4 mM glutamine (PanEco, Moscow, Russia). After 3-day culturing in a 96-well plate in an incubator at 37 °C, 5% CO_2_, the culturing medium was changed and scaffold samples were brought to the wells: one 5 × 5 mm film sample per well. Scaffold samples were also incubated for 7 days at 37 °C, 5% CO_2_ conditions, while the culture plastic was used as a control. Then, each well was supplied with 60 μL of a 5 mg/mL methylthiazolyldiphenyl-tetrazolium bromide (MTT; Dia-M, Moscow, Russia) solution, which was incubated for 4 h at 37 °C, 5% CO_2_, followed by precipitation of dark blue formazan crystals. After removal of scaffold samples, the plate was centrifuged at 885 g for 5 min. The formazan precipitates were dissolved in 300 μL of dimethyl sulfoxide (Panreac, Barcelona, Spain) for 20 min, and the solution optical density at a wavelength of 540 nm was measured with a Picon spectrophotometer (Uniplan, Picon Co., Moscow, Russia) for each well. The experiment was conducted on 5 samples of each silk fibroin scaffold and composite scaffold group, and the experiment was conducted in duplicate.

### 2.9. Cell Proliferation

Cell proliferation on fabricated scaffolds was studied using two standard cell lines: the mouse 3T3 fibroblasts (ATCC, CRL 1658) and the Hep-G2 human hepatocarcinoma cells (ATCC, HB-8065). Both cell lines were obtained from Dr. M.E.Krasheninnikov (People’s Friendship University of Russia, Moscow, Russia). Scaffolds of both groups were placed in the wells of the 96-well plate, while culture plastic was used as a control. The cell suspension in the medium was transferred into 96-well plates at the rate of 1000 cells per well. Cells were incubated for 7 days at 37 °C and 5% CO_2_. 3T3 line fibroblasts were incubated in DMEM Low glucose medium, while Hep-G2 cells were incubated in DMEM High glucose (PanEco, Moscow, Russia) and Ham’s F-12 (PanEco, Moscow, Russia) mixed culture media; all media contained 10% fetal bovine serum, 10 mg/mL gentamicin solution, and 0.324 mg/mL glutamine.

The samples were stained with DAPI (Sigma-Aldrich, St. Louis, MO, USA) fluorescence dye, and 3 µg aqueous DAPI solution was added at a rate of 150 µL per well and incubated for 5 min at 37 °C, 5% CO_2_. Then, the samples were washed twice with PBS.

Cell adhesion and proliferative activity were investigated by fluorescence optical microscopy using a Carl Zeiss Axio Vert.A1 microscope (Carl Zeiss AG, Jena, Germany) with an objective lens LD Plan-Neofluar 20x/0.4 Corr M27 (Carl Zeiss AG, Jena, Germany) and a filter cube adjusted for DAPI dye, having an excitation range of 360–370 nm, an emission range of 420–470 nm. Images of cells in the 425 × 350 µm field of view were obtained with an Axiocam 305 color camera (Carl Zeiss AG, Jena, Germany) and processed by the Zen 2.3 Blue Edition program (Carl Zeiss AG, Jena, Germany). Quantity of cells for each sample was obtained by direct counting of DAPI-stained nuclei in the obtained images. The experiment was conducted on 5 samples of each experimental group, and the experiment was conducted in duplicate.

### 2.10. In Vivo Analysis of Composite Scaffold’s Regenerative Potential

An in vivo experiment was performed on a model of the rat skin wound healing. The animals were divided into three groups: group 1—control group, where the wounds were not covered with any material or construction that promoted skin healing; group 2—experimental group, where the wounds were covered with scaffolds in the form of films based on silk fibroin; group 3—experimental group, where the wounds were covered with composite scaffolds in the form of films based on silk fibroin with microparticles of decellularized rat liver tissue. There were 5 rats in each group. Ether was used for inhalation anesthesia at the rate of 50 mg/kg for all surgery manipulations with animals, which were in spontaneous respiration. Full-thickness wounds of circular shape were cut with surgical scissors on the back in the area of the rat shoulder after hair removal. Average diameter of wounds was 14 mm. An aqueous solution (0.05%) of chlorhexidine bigluconate (RosBio, Saint-Petersburg, Russia) was used for wound treatment. Sterile gauze dressing was utilized for covering the wounds of control group rats. Photographs of wounds were acquired on the 3rd, 9th, 14th, 18th, and 23rd day of the experiment for the experimental group, and on the same days plus the 28th, 35th, 40th day for the control group, with a Nikon D5100 18-105 VR Kit camera with a Nikon AF-S DX 18–105 mm f/3.5–5.6 G ED VR lens (Nikon, Tokyo, Japan). The progress of skin wound healing was assessed quantitatively using a wound-healing area parameter, calculated according to the formula:

(1) $$A\;=\;\frac{d_{\left(0\right)}-d_{\left(t\right)}}{d_{\left(0\right)}}\;\times \;100\%.$$

where A is the wound healing area, $d_{\left(0\right)}$ is the initial wound diameter (day 0), $d_{\left(t\right)}$ is the wound diameter on the corresponding experiment day (t).

Obtained quantitative data enabled us to plot healing diagrams of skin wounds in all three groups. After complete healing of a full-thickness skin wound was confirmed visually, samples of skin tissue with dimensions of 20 × 20 mm were collected for consequent histological analysis.

### 2.11. Histological Analysis

Samples of skin tissue were fixed using a mixture of formalin, ethanol, and acetic acid in a volume ratio of 4:1:0.3, incubated into paraffin for 48 h at room temperature, and embedded in paraffin. Then, embedded samples were sectioned with a Thermo Scientific HM 325 Rotary Microtome (Thermo Fisher Scientific, Waltham, MA, USA) and sections of 14 μm thickness were collected. All sections were stained with Masson’s trichrome stain (Sigma-Aldrich, USA), embedded in Canada balsam, and studied with a Carl Zeiss Axio Vert.A1 optical microscope (Carl Zeiss AG, Jena, Germany) with an Axiocam 305 color camera (Carl Zeiss AG, Jena, Germany). Images were processed using Zen 2.3 Blue Edition software (Carl Zeiss AG, Jena, Germany).

### 2.12. Statistical Analysis

The data were processed by ANOVA. The statistical significance of differences in the results was evaluated by the Mann–Whitney U test. The level of statistical significance α was considered equal to 0.05.

## 3. Results

**Figure 1:**
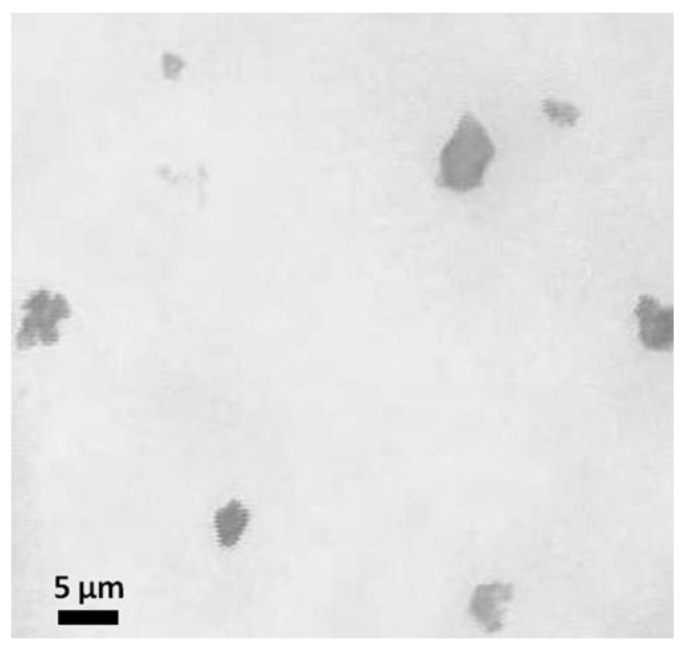
Image of microparticles of extracellular matrix of decellularized rat liver (×100, phase contrast).

Liver tissue microparticles were obtained by grinding the decellularized matrix in liquid nitrogen. The fraction of microparticles of the rat liver extracellular matrix with an average size of 1–5 μm was 20% by weight of the total amount of tissue (Figure 1).

**Figure 2:**
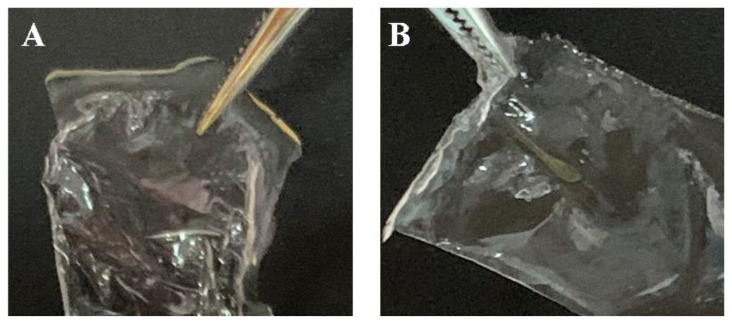
Scaffolds based on silk fibroin. (**A**) Scaffold in the form of a film based on silk fibroin (SF), (**B**) scaffold in the form of a film based on silk fibroin, with microparticles of decellularized rat liver tissue included in the composition (SF-MP).

Scaffolds in the form of films based on silk fibroin with microparticles of decellularized rat liver tissue included in the composition were made by the casting method (Figure 2). Two groups of samples were prepared: group 1—scaffolds in the form of films based on silk fibroin, referred to as SF; group 2—composite scaffolds in the form of films based on silk fibroin with microparticles of decellularized rat liver tissue, referred to as SF-MP. All obtained scaffolds were transparent; the inclusion of microparticles in the scaffold’s composition did not affect the transparency of the constructions. Composite scaffolds were obtained by mixing a silk fibroin solution with a suspension of microparticles to a final particle concentration of 8 mg/mL; the obtained solution was applied to the surface of polished Teflon and dried at room temperature.

**Figure 3:**
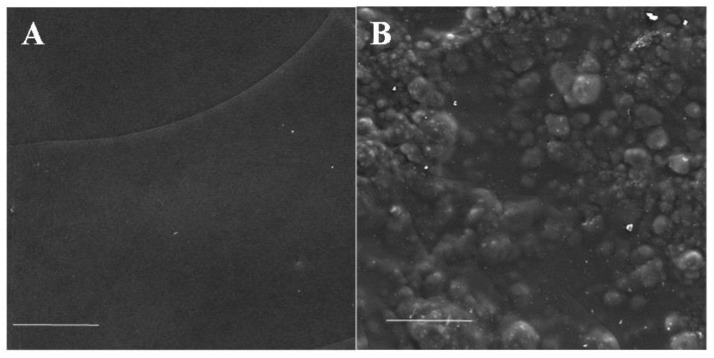
Image of the surface of scaffold samples obtained by scanning electron microscopy. (**A**) SF scaffold, (**B**) SF-MP scaffold. Scale bar—10 µm.

**Figure 4:**
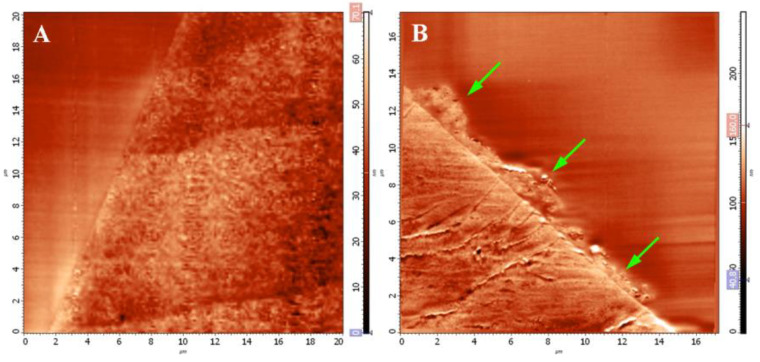
SPM images of the surface of the cross-section of the scaffold in the form of a film. (**A**) SF scaffold (scanning area 20 × 20 μm), (**B**) SF-MP scaffold (scanning area 17 × 17 μm). Microparticles of decellularized tissue are indicated by arrows.

The surface structure of the obtained composite scaffolds was analyzed by scanning electron microscopy. The surface topography of the scaffold, with included microparticles, was rougher and non-uniform compared with the surface topography of a non-composite scaffold (Figure 3). The micro- and nanostructure of the surface of the obtained scaffolds, as well as the structure of microparticles in the composition of the scaffolds, were studied by scanning probe microscopy. Figure 4 shows the SPM images of the surface topography of the epoxy-embedded scaffold’s cross-section surface prepared by the ultramicrotomy.

Microparticles of decellularized tissue were observed on the surface of the composite scaffolds, in addition to microparticles located in the volume of the film. At the same time, microparticles of decellularized rat liver tissue were spread over the surface of the scaffold and created a rough relief of the film surface.

Based on the obtained SPM images, a quantitative analysis of the roughness of the studied scaffolds was conducted. Data on the roughness of the scaffold surfaces are presented in Table 1.

The data presented in the table indicated that the inclusion of the extracellular matrix of the liver tissue in the composition of the microparticles increased the surface roughness of the construction five-fold.

**Figure 5:**
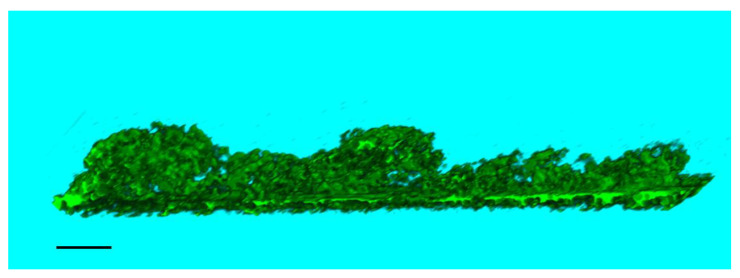
Visualization of three-dimensional model of the microparticles of decellularized rat liver tissue on the surface of a SF-MP scaffold reconstructed by SPNT technique: reconstructed volume 22.40 × 4.70 × 2.25 μm, number of sections 15, section thickness 150 nm, scale bar 2 μm.

The analysis of the micro- and nanostructure of the obtained composite construction was conducted by the SPNT method. For quantitative analysis of the construction, three-dimensional reconstructions were created by combining a series of SPM-sections of scaffolds (Figure 5), depicting microparticles of decellularized rat liver tissue on the surface of the scaffold.

The three-dimensional reconstructions were obtained to calculate the ratio of the volume of microparticles to the volume of the scaffold. For a scaffold in the form of a film based on silk fibroin, with microparticles of decellularized rat liver tissue included in the composition, this ratio was 1.4% ± 0.4.

A study of the effect of inclusion of microparticles of decellularized tissue into the composition of scaffolds in the form of films based on silk fibroin on the mechanical properties of the constructions was conducted. The samples were subjected to deformation on a Zwick/Roell BZ 2.5/TNIS tensile testing machine, and the values of the material’s tensile strength (MPa) and elasticity (% elongation) were evaluated (Table 2). According to the experimental analysis of mechanical properties, the inclusion of decellularized liver tissue such as that of rats in the composition of microparticles did not affect the elasticity and tensile strength of the scaffolds.

**Figure 6:**
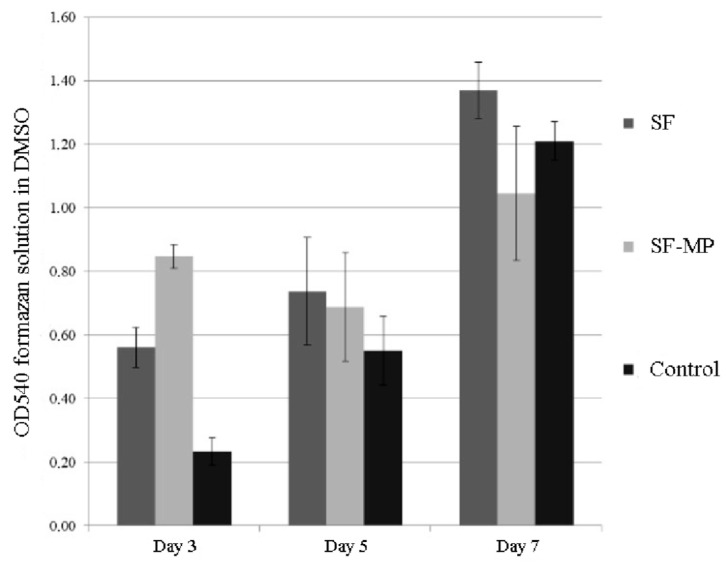
Data on cytotoxicity of scaffolds based on silk fibroin. Standard deviation values for five independent measurements are shown.

Analysis of the cytotoxicity of the obtained scaffolds was conducted using the 3T3 mouse fibroblast cell line. During the experiment, samples of two types of scaffolds were placed next to a monolayer of mouse fibroblasts and incubated in a culture medium for 7 days. The obtained scaffolds were not toxic to the 3T3 mouse fibroblast cells. (Figure 6).

**Figure 7:**
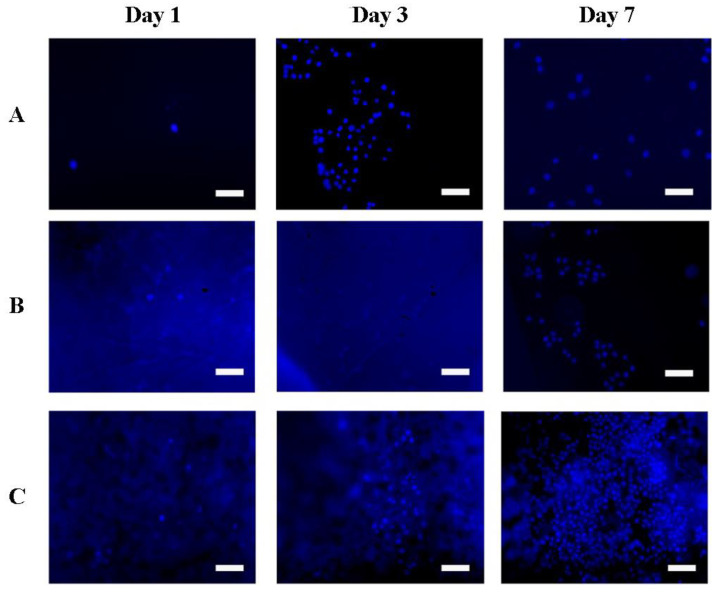
Images of 3T3 mouse fibroblast cells cultured on the obtained scaffolds. The scale bar is 100 µm. Cell nuclei are stained with DAPI. (**A**) control (cultural plastic), (**B**) SF scaffold, (**C**) SF-MP scaffold.

**Figure 8:**
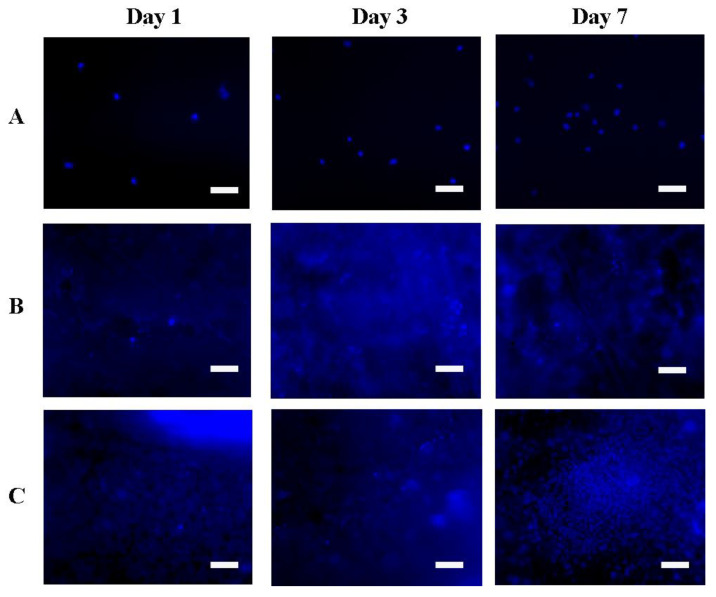
Images of Hep-G2 cells cultured on the obtained scaffolds. The scale bar is 100 µm. Cell nuclei are stained with DAPI. (**A**) control (cultural plastic), (**B**) SF scaffold, (**C**) SF-MP scaffold.

Adhesion and proliferation of 3T3 mouse fibroblasts and Hep-G2 human hepatocarcinoma cells were studied on samples of the obtained scaffolds in the form of films based on silk fibroin. Culture plastic was used as a control. Fluorescent microscope images of the number of cells and their distribution on the scaffolds during the experiment are shown in Figure 7 and Figure 8.

**Figure 9:**
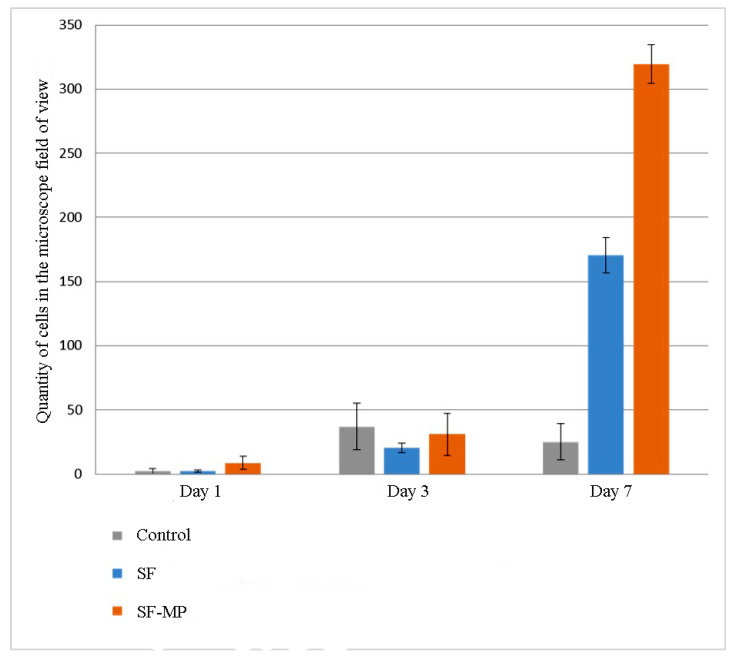
Data on the proliferative activity of 3T3 culture cells on days 1, 3, and 7 of the experiment on the obtained scaffolds based on silk fibroin. Standard deviation values for five independent measurements are shown.

**Figure 10:**
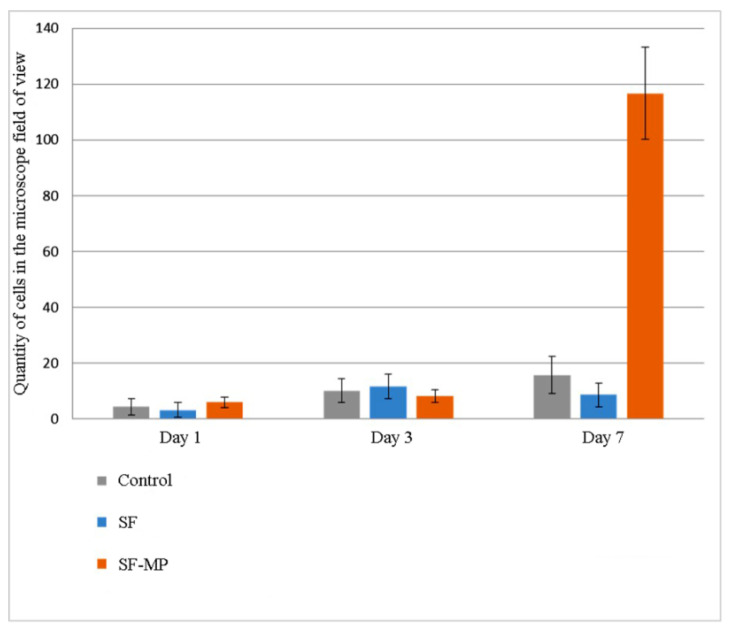
Data on the proliferative activity of Hep-G2 culture cells on days 1, 3, and 7 of the experiment on the obtained scaffolds based on silk fibroin. Standard deviation values for five independent measurements are shown.

Quantitative results of proliferative activity analysis of 3T3 and Hep-G2 culture cells are depicted in Figure 9 and Figure 10 correspondingly. It was shown that all scaffolds based on silk fibroin supported cell adhesion; there were no significant differences between different groups of samples and control. Furthermore, during the experiment to assess proliferation, the largest number of cells on the 7th day of the experiment of both cultures was observed on scaffolds in the form of a film based on silk fibroin, with microparticles of decellularized rat liver tissue included in the composition. On composite scaffolds, the proliferative activity of cells on the 7th day of observation was higher than on unmodified scaffolds.

**Figure 11:**
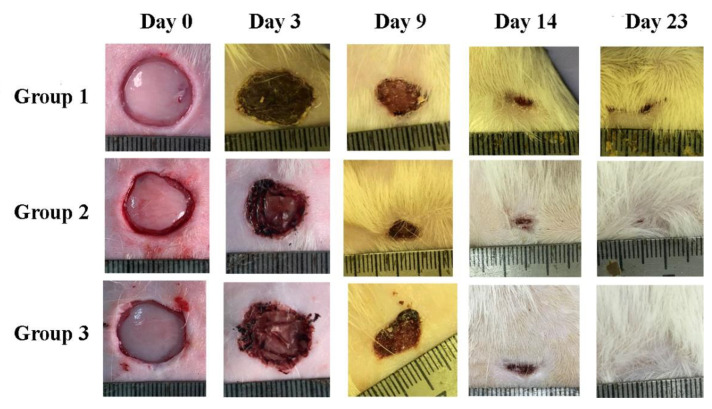
Images of wounds on days 0, 3, 9, 14, and 23 of the experiment. Group 1: control, group 2: SF, and group 3: SF-MP.

The fabricated composite scaffolds with microparticles of decellularized rat liver tissue included in the composition were successfully used as wound dressings for the full-thickness rat skin wound-healing model. Images of wounds during the experiment are shown in Figure 11.

**Figure 12:**
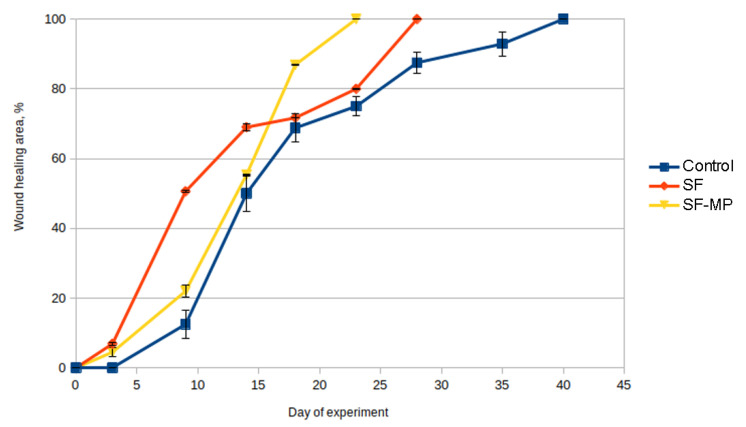
Wistar rat skin healing curves.

To quantify the wound-healing process, wound diameters were measured on the 0th, 3rd, 9th, 14th, 18th, 23rd, 28th, 35th, and 40th days of the experiment (Table 3), and healing curves were plotted (Figure 12).

All scaffolds were hydrophilic, so they were easily positioned on the wound and did not require additional fixation with suture material. According to the obtained results, an increase in the rate of wound healing in the experimental group was observed after 9 days. Complete wound healing occurred in group 3 on day 23, and in group 2 on day 28, while in the control group complete wound closure was observed at day 40 (Table 3, Figure 12).

**Figure 13:**
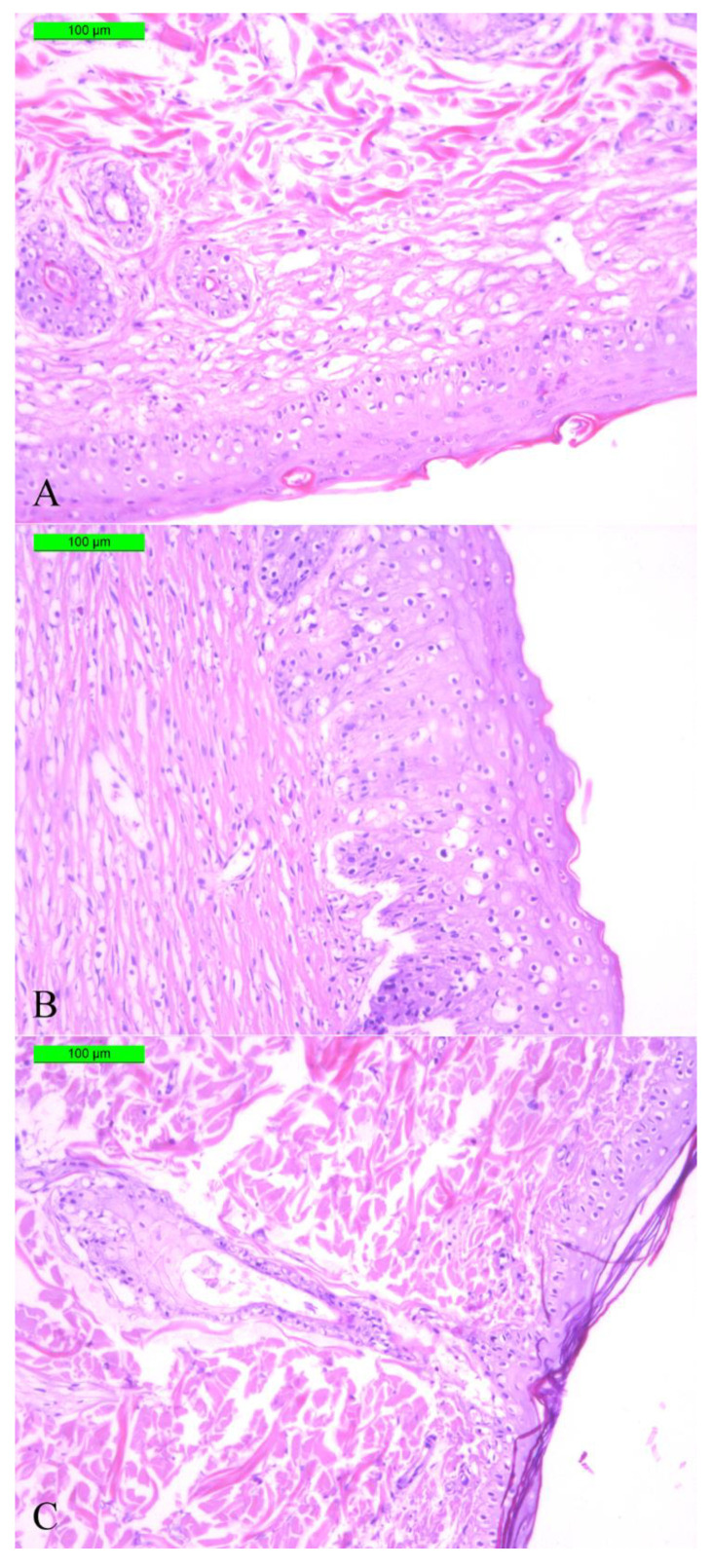
Optical microscopy images of skin tissue sections stained by Masson’s trichrome. (**A**) Control (40 days), (**B**) wound covered with SF scaffold (28 days), (**C**) wound covered with SF-MP scaffold (23 days). Scale bar 100 µm.

After complete wound healing on the aforementioned days of the experiment for each group, sections of skin tissue samples from the wound areas were collected from all three groups, stained with Masson’s trichrome, and studied by optical microscope. Thus, we compared completely regenerated skin tissue in the control group on day 40 with samples of regenerated skin tissues from the experimental groups from considerably earlier periods to prove the observed acceleration of full skin regeneration in the experimental groups by histology means. A histological evaluation of skin samples in the area of complete wound healing for each group (A = 100%) revealed the restoration of three layers of skin in all the experimental animals—epidermis, dermis, and hypodermis—which indicated successful wound healing (Figure 13). Moreover, as seen in Figure 13C, a hair follicle was observed, which also indicated a complete functional restoration of the skin. Figure 13B reveals a thicker stratum corneum of the epidermis and a friable structure of the dermis. A looser structure of the dermis relative to the control was also observed on histological sections of samples of group 2.

## 4. Discussion

A main approach of tissue engineering and regenerative medicine in the restoration of organ and tissue pathological injuries is the fabrication of construction that mimics the structure of tissues and organs. The relevant selection of the materials is a crucial parameter for the manufacturing of such constructions. It is essential to use materials that combine the properties to provide both high regeneration efficiency and the possibility of implantation into the body.

Constructions that are made of synthetic materials, such as polycaprolactone, polyglycolide, and polyamide, generally have good mechanical properties, allowing them to adjust their shape, structure, and physical parameters, but their degradation products can be toxic to cells.

The utilization of natural materials, which are able to mimic the microenvironment of native tissue as closely as possible, is associated with a number of difficulties. These constructions are obtained from native tissues, which implies high costs, ethical issues, and low mechanical properties of obtained grafts. This leads to the emergence of prerequisites for the development of new constructions for regenerative medicine.

One promising material for the creation of biocompatible constructions for tissue engineering and regenerative medicine is silk fibroin from the cocoons of the silkworm *Bombyx mori* [38]. The structure of silk fibroin provides unique favorable mechanical properties with a high level of biocompatibility, allowing utilization of this polymer as a material for bioartificial constructions. In addition, silk fibroin is a non-immunogenic material: the absence of an immune response to silk fibroin constructions has been shown in various in vivo and in vitro models [39]. It was also shown that silk fibroin has antibacterial properties: the adhesion of bacterial cells to silk fibroin constructions is significantly reduced compared with other materials, owing to the physicochemical properties of silk fibroin. It should be noted that grafts based on silk fibroin can be obtained under mild conditions. Thus, fibroin has unique properties that allow forming two-dimensional and three-dimensional constructions and widely utilizing it as a biocompatible material in various areas of tissue engineering. The biocompatibility of silk fibroin constructions can be improved by incorporating microparticles of the extracellular matrix of native tissue into the construction. This modification will allow recreating a native microenvironment for cells in a bioartificial matrix while retaining the mechanical properties of constructions based on silk fibroin only. The utilization of composite constructions will facilitate manipulations comparable to fragments of decellularized tissue and will facilitate the positioning of extracellular matrix fragments during implantation.

Within our framework, scaffolds in the form of films based on silk fibroin with microparticles of decellularized rat liver tissue were fabricated by the casting method. Microparticles were obtained by grinding decellularized liver tissue in liquid nitrogen. All scaffolds were colorless, transparent, and had a thickness of 20 to 60 µm.

The surface structure of the obtained scaffolds was studied with SEM. The surface of the composite scaffolds had a rough structure compared to the unmodified scaffold. Moreover, the surface topography of the composite scaffold was uneven over the entire surface area with distinct local clusters of microparticles.

The micro- and nanostructure of the surface of the obtained scaffolds, as well as the structure of the microparticles in the scaffold composition, were studied with scanning probe microscopy. It was shown that microparticles of decellularized tissue were present both in the thickness of composite scaffolds and on their surface. Thus, it can be concluded that the chosen method of manufacturing composite scaffolds was effective, while some microparticles were not shielded by silk fibroin, which can affect the interaction of cells with the construction before the scaffold degradation begins. In the quantitative analysis of SPM images, it was shown that the inclusion of liver tissue in the microparticles of the extracellular matrix in the scaffold composition increased the surface roughness of the construction by 5 times compared with non-composite scaffolds. Substrate roughness is known to be an important parameter that determines the level of cell adhesion, proliferation, and differentiation [40,41].

The study of the micro- and nanostructure of the obtained composite scaffolds was conducted with the SPNT method. It was shown that rat liver microparticles were flattened on the scaffold surface and had a large area of contact with it. The unique capabilities of the SPNT method made it possible to identify the morphology of the contact between the microparticle and the scaffold core, which was impossible using other microscopy methods, including SEM [37,42].

Important properties of materials and constructions for regenerative medicine are the mechanical characteristics, which allow performing surgical procedures during implantation of the obtained constructions. Constructions based on *Bombyx mori* silk fibroin aqueous solution had tensile strength and elasticity comparable to products actively used in regenerative medicine [43]. At the same time, the inclusion of decellularized tissue microparticles in the composition did not affect the elasticity and tensile strength of the scaffold in the form of a film and did not affect the biodegradation rate of the scaffold in two media: neutral and oxidizing. However, during the biodegradation analysis, washout of microparticles was observed.

Furthermore, a study of the biocompatibility and an assessment of the regenerative potential in vivo of the obtained scaffolds in the form of films were conducted. Before conducting these experiments, an analysis of the cytotoxicity of the scaffolds was conducted, which did not reveal a cytotoxic effect on the cells of the obtained scaffolds based on silk fibroin.

The biocompatibility of the constructions was assessed using two cell cultures, 3T3 mouse fibroblasts and Hep-G2 human hepatocarcinoma cells. During the experiment, it was shown that in all scaffolds based on silk fibroin support cell adhesion, there were no significant differences between different groups of samples and control. On composite scaffolds, the proliferative activity of cells on the 7th day of observation was higher than on unmodified scaffolds, which may be because of the optimal roughness of composite constructions. The largest number of cells of both cultures on the 7th day of the experiment was registered on scaffolds in the form of a film based on silk fibroin, with microparticles of decellularized rat liver tissue included in the composition. This may have depended on the shape and position of decellularized tissue microparticles on the scaffold surface; they were spread over the surface of the construction, which created an optimal relief and substrate roughness for cell adhesion and proliferation. This may also have been connected with the tissue-specific properties of the microparticles of decellularized tissue [44] or due to the biochemical composition of microparticles exposed on the film’s surface. According to a previous study [35], the biochemical composition of the extracellular matrix after liver decellularization remained, including different types of collagens, which play a crucial role in the adhesion and proliferation of cells owing to RGD sequences in their structure.

The regenerative potential and the possibility of application of composite scaffolds based on silk fibroin were studied in a full-thickness rat skin wound-healing model.

All obtained scaffolds were successfully implanted. Scaffolds based on silk fibroin were hygroscopic, fixed on the wound without suture material, retaining moisture throughout the entire procedure [45]. Scaffolds were easily positioned on the wound; the possibility of not having to sew on the construction made the procedure less invasive and traumatic. During manipulations of the procedure, no differences were found in working with the obtained scaffolds.

Skin healing was quantified by measuring the diameter of wounds during the experiment. Scaffolds based on silk fibroin of both groups accelerated wound healing compared with controls.

The shape of the curve of the dynamics of wound healing with composite scaffolds as wound dressing did not differ from the shape of the wound healing curve of the control. The utilization of scaffolds based on silk fibroin with microparticles of decellularized tissue accelerated wound healing by 17 days (1.74 times) compared with the control, and by 5 days (1.22 times) compared with the unmodified scaffold.

An investigation of the quality of the tissue restoration in the full-thickness skin wounds was performed by histological analysis of sections of skin tissue samples obtained after complete healing of the wounds. The study indicated that all three layers of skin tissue were presented, similar to the native skin, indicating successful regeneration. The sections from the experimental group using an SF scaffold demonstrated a more pronounced stratum corneum of the epidermis and a loose structure of the dermis. Importantly, no macrophage presence or inflammation foci were observed, which also proved the quality of skin healing. The presence of hair follicles in groups of composite scaffolds was shown, which also indicated a complete functional restoration of the skin.

It makes sense to compare observed acceleration of skin regeneration with the results obtained previously for other scaffolds studied in the similar full-thickness rat skin wound-healing model, namely electrospun microfibrous scaffolds based on silk fibroin [36] and lyophilized decellularized Wistar rat liver fragments (LDLFs) [35].

Silk fibroin-based microfibrous scaffolds demonstrated a regenerative effect analogous to SF-MP scaffolds studied in the present work [36], while LDLFs performed even better, accelerating wound healing by 22 days (2.2 times) in comparison with the control [35]. However, microfibrous scaffolds need more sophisticated fabrication techniques and are much more vulnerable for mechanical damage in surgical operations than silk fibroin-based scaffolds in the form of films. LDLFs are limited in size (we used 2 × 2 mm fragments) and require a significantly (at least 100 times) greater amount of the liver tissue for fabrication than SF-MP scaffolds considering equivalent wound area. Silk fibroin-based scaffolds in the form of films with microparticles of decellularized tissue can be produced in almost any shape and size reasonable for biomedical applications.

Thus, it can be concluded that the obtained scaffolds based on silk fibroin with microparticles of decellularized rat liver tissue have high regenerative potential in vitro and in vivo and may be considered as a basis for development of new biomedical products for regenerative medicine.

## Figures and Tables

**Table 1:** Data on the average surface roughness of the obtained scaffolds. Standard deviation values for six independent measurements are shown.

Type of Scaffold	The Average Surface Roughness, nm
SF	36.5 ± 10.6
SF-MP	195.0 ± 40.0

**Table 2:** Measurement data of tensile strength and elasticity of various samples of scaffolds based on silk fibroin. Standard deviation values for five independent measurements are shown.

Type of Scaffold	Tensile Strength, MPa	Elasticity Elongation, %
SF	81.3 ± 12.6	37.2 ± 9.7
SF-MP	86.4 ± 10.5	33.8 ± 7.4

**Table 3:** Dynamics of full-thickness skin wound healing in Wistar rats (standard deviation values for five independent measurements are shown).

Group No.	Day 0	Day 3	Day 9	Day 14	Day 18	Day 23	Day 28	Day 35
1	0	0	12.5 ± 4.0	50.0 ± 5.1	68.8 ± 4.0	75.0 ± 2.7	87.5 ± 3.1	92.9 ± 3.5
2	0	6.9 ± 0.5	50.6 ± 0.3	69.0 ± 1.0	71.7 ± 0.1	80 ± 0.1	100	
3	0	4.5 ± 1.2	22.0 ± 1.8	55.4 ± 0.1	86.9 ± 0.2	100	100	

## Data Availability

The data presented in this study are available on request from the corresponding authors.
